# The role of ımmune cells in the placenta of gestational diabetes patients: does ıt offer hope for targeted treatment?

**DOI:** 10.1007/s00404-025-08289-9

**Published:** 2026-01-07

**Authors:** Denizhan Bayramoğlu, Celal Akdemir, Sibel Özler, Zeynep Bayramoğlu

**Affiliations:** 1 Department of Obstetrics and Gynaecology, Division of Gynaecological Oncology, İzmir City Hospital, İzmir, Türkiye; 2 Department of Obstetrics and Gynecology, Division of Perinatology, Faculty of Medicine, KTO Karatay University, Medicana Hospital, Konya, Türkiye; 3 Department of Pathology, Faculty of Medicine, Dokuz Eylul University, Izmir, Türkiye; 4 Department of Molecular Pathology, Faculty of Health Sciences, Dokuz Eylul University, Izmir, Türkiye

**Keywords:** Gestational diabetes mellitus, Placenta, Immunohistochemistry, CD4, Positive T, Lymphocytes, CD8, Positive T, Lymphocytes, Macrophages, Inflammation

## Abstract

**Background:**

Gestational diabetes mellitus (GDM) is a prevalent metabolic complication that arises during pregnancy, posing significant health risks for both mother and fetus. The placenta is not only affected by GDM but also actively contributes to its pathogenesis and maternal–fetal outcomes. This complex interaction makes it difficult to fully understand the etiology of GDM and its effects on the placenta. In this study, we aimed to clarify the pathogenesis of GDM by evaluating the role of inflammation and describing the macroscopic and histopathological changes in placentas affected by GDM.

**Methods:**

This study compared 50 singleton pregnancies complicated by GDM with 50 normoglycemic pregnancies. All deliveries occurred at term. Placentas were examined both macroscopically and microscopically. Immunohistochemical staining was performed for the following markers: CD4, CD8, CD68, CD80, CD86, and CD206.

**Results:**

Placental weight and diameter were significantly higher in the GDM group compared to the control group (p < 0.001). GDM placentas showed a significantly higher frequency of chorangiosis, villous edema, villous immaturity, and ischemic changes (p < 0.001). Immunohistochemical analysis revealed increased expression of CD4, CD8, CD68, CD80, and CD86, while CD206 expression was significantly reduced in the GDM group (p < 0.001).

**Discussion:**

These findings support the central role of placental inflammation and macrophage polarization shifts in the pathogenesis of GDM. They also highlight potential targets for developing new diagnostic biomarkers and anti-inflammatory or immunomodulatory therapeutic strategies.

## What does this study add to the clinical work?

**Table:** 

This study demonstrates that gestational diabetes is associated with significant inflammatory and histopathological alterations in the placenta, accompanied by shifts in immune cell profiles and macrophage polarization. These findings support the role of placental inflammation in the pathogenesis of gestational diabetes and may provide a basis for future diagnostic and immunomodulatory therapeutic approaches.	

## Introduction

Gestational diabetes mellitus (GDM) is a common metabolic disorder that develops during pregnancy and poses both short- and long-term health risks for the mother and the fetus [1]. GDM is characterized by glucose intolerance, usually diagnosed in the second or third trimester, resulting from increased insulin resistance and relative insulin insufficiency. Women diagnosed with GDM are at a significantly higher risk of developing type 2 diabetes later in life.

Gestational diabetes mellitus (GDM) is a growing global health concern, with a pooled standardized prevalence of approximately 14.0% (95% CI 13.97–14.04%) across populations, according to the International Diabetes Federation (IDF) and recent meta-analyses. Regional variation is considerable—from around 7.1% in North America and the Caribbean to as high as 27.6% in the Middle East and North Africa (MENA) (2). In Turkey, a recent systematic review and meta-analysis covering 50,767 pregnancies estimated an overall GDM prevalence of 7.7%, ranging between 1.9 and 27.9% across regions, with the highest rates in the Black Sea region (17.6%) and the lowest in Central Anatolia (5.1%) (3). These figures highlight the substantial and regionally variable burden of GDM, emphasizing the need for improved screening, preventive strategies, and mechanistic studies to better understand immune and metabolic interactions during pregnancy.

Recent studies have increasingly highlighted the central role of chronic, low-grade inflammation in the pathophysiology of GDM. This inflammatory state is associated with elevated levels of pro-inflammatory cytokines such as tumor necrosis factor-alpha (TNF-α), interleukin-6 (IL-6), and interleukin-8 (IL-8) in circulation and tissues. Moreover, oxidative stress is elevated in GDM and interacts closely with inflammation [6].

During pregnancy, the immune system undergoes crucial adaptations to support fetal development and maintain maternal tolerance. Lymphocytes and tissue macrophages are key immune cells involved in this process. The placenta houses a specialized subset of macrophages known as Hofbauer cells, which play a central role at the maternal–fetal interface in maintaining immune tolerance [7]. In GDM, alterations have been observed in both circulating monocytes and Hofbauer cell properties. Hyperglycemia and changes in Hofbauer cells are also associated with altered CD4 and CD8 T cell profiles, suggesting increased placental inflammation. Inflammation is closely linked to other pathological processes in GDM, including disrupted fatty acid metabolism and placental angiogenesis.

The complex etiology of GDM and tissue-specific immune responses complicate the full elucidation of its mechanisms. Therefore, a detailed examination of immune cells and specific markers in GDM placentas is crucial for identifying potential diagnostic and therapeutic targets. In this study, we aimed to investigate placental inflammation and characterize macroscopic and histopathological changes in GDM placentas to shed light on the pathogenesis of this condition.

## Materials and methods

### Study design and participants

This study was approved by the ethics committee of the university of health sciences (Approval No: 20/313, dated September 11, 2020), and written informed consent was obtained from all participants. A total of 100 singleton pregnancies were included, comprising 50 cases of GDM diagnosed between 28 and 35 weeks of gestation and 50 normoglycemic pregnancies serving as the control group. All deliveries occurred at term (between 37 and 41 weeks of gestation).

Routine ultrasonographic evaluations were performed between 23–24 and 33–36 weeks of gestation to confirm normal fetal growth. GDM was diagnosed between 24 and 32 weeks of gestation using the 100 g, 3 h oral glucose tolerance test (OGTT) according to the 2000 American Diabetes Association (ADA) criteria. These criteria were applied as they represent the institutional standard at our center and ensure methodological consistency across the study period. For context, more recent international recommendations (IADPSG 2010; WHO 2013) employ a one-step 75 g OGTT with lower diagnostic thresholds, but these were not yet adopted during the data collection period of this study [8, 9].

Pregnancies complicated by other conditions (e.g., preeclampsia, eclampsia), chronic diseases, tobacco or alcohol use were excluded from the study. Gestational age was determined based on the last menstrual period and/or ultrasound findings. If there was more than a one-week discrepancy between the menstrual date and ultrasound, first-trimester ultrasonography was used to confirm gestational age.

## Gross and histopathological evaluation

Placental specimens were first examined macroscopically for umbilical vascular abnormalities. Each placenta was weighed and measured in three dimensions. Samples were collected from the umbilical cord, cord insertion site, and placental membranes. Additionally, at least three full-thickness tissue sections were taken from areas that appeared grossly normal and abnormal (e.g., hemorrhagic or solid lesions). Tissue samples were collected immediately after delivery, fixed in formalin, embedded in paraffin blocks, and stained with hematoxylin and eosin (H&E). Histopathological evaluations focused on the presence of villous immaturity, chorangiosis, ischemia, and hydropic villi.

## Immunohistochemistry

Formalin-fixed, paraffin-embedded placental tissue sections were processed using the Ventana Benchmark Ultra fully automated immunohistochemistry system (Roche Diagnostics, Basel, Switzerland) according to standard manufacturer protocols. The following immune markers were analyzed semi-quantitatively: CD4 (clone SP35, Roche), CD8 (clone SP57, Roche), CD68 (clone KP1, Roche), CD80 (clone EP1158Y, Roche), CD86 (clone EP1158Y, Roche), and CD206 (clone EPR22485-161, Roche). Immunoreactivity was quantified as the average number of positively stained cells per ten high-power fields (HPFs) in representative placental regions.

## Statistical analysis

Continuous variables were summarized as mean ± SD and median (IQR). Normality was assessed using the Shapiro–Wilk test and homogeneity of variances using Levene’s test. Between-group comparisons were performed using Welch’s t test when distributional assumptions were met or variances were unequal; otherwise, the Mann–Whitney U test was used. Effect sizes were reported as Cohen’s d for parametric contrasts and rank-biserial correlation (r_rb) for non-parametric tests. To control for multiple testing across immune markers, a Bonferroni correction was applied (adjusted α = 0.008). Two-tailed p < 0.05 was considered statistically significant.

## Correlation analysis and heatmap visualization:

To evaluate the relationships among continuous variables, pairwise correlation coefficients were calculated using the Pearson correlation method. Pearson’s correlation assesses the strength and direction of the linear relationship between two variables. The resulting correlation matrix was visualized as a heatmap using the seaborn.heatmap function. This graphical representation provided an intuitive overview of correlation magnitudes, with color gradients indicating the strength and direction of associations (ranging from − 1 to + 1).

## Results

A total of 100 placentas were examined, comprising 50 from normoglycemic pregnancies (control group) and 50 from pregnancies complicated by gestational diabetes mellitus (GDM). A comparative analysis was performed between the two groups focusing on placental morphometry, histopathological alterations, and immune marker expression. The demographic and baseline clinical characteristics of the study participants are summarized in Table 1. No significant differences were observed between the GDM and control groups regarding maternal age, gestational age at delivery, gravidity, parity, or pre-pregnancy BMI (all p > 0.05). These findings indicate that both groups were comparable in terms of major obstetric parameters, ensuring the validity of subsequent immunological comparisons.

**Table 1:** Demographic and baseline clinical characteristics of pregnant women with GDM and controls (Data are presented as mean ± SD and median (IQR). p values derived from Mann–Whitney U tests)

Variable	GDM + mean ± SD	GDM − mean ± SD	p	Cohen’s d	Interpretation
Age [years]	32.69 ± 4.83	31.08 ± 3.47	0.325	0.38	Small–medium effect
Gestational age [weeks]	38.31 ± 1.31	39.88 ± 1.07	0.262	−1.28	Large effect (favors control)
Gravidity	2.19 ± 1.36	2.97 ± 1.53	0.153	−0.55	Medium effect (favors control)
Parity	1.41 ± 0.48	1.88 ± 0.86	0.710	−0.68	Medium effect (favors control)
Initial BMI [kg/m^2^]	21.55 ± 3.14	21.67 ± 3.54	0.978	−0.04	Negligible

## Placental morphometry

The mean placental weight was significantly higher in the GDM group compared to the control group (898.0 g vs. 718.0 g, p < 0.001; Mann–Whitney U test). Similarly, the mean maximum placental diameter was greater in GDM cases (19.88 cm vs. 17.44 cm; p < 0.001).

## Histopathological findings

Histological examination revealed a markedly increased incidence of specific pathological features in the GDM group:

- **Chorangiosis** was observed in 36 GDM placentas compared to 7 in the control group (p < 0.001; Chi-square test),
- **Villous edema** occurred in 26 GDM vs. 10 control placentas (p < 0.001),
- **Villous immaturity** was identified in 29 GDM cases vs. 16 controls (p < 0.001),
- **Ischemic changes** were present in 29 GDM placentas compared to 16 in the control group (p < 0.001).

These findings indicate that GDM is not only associated with increased placental size but also with a higher frequency of histopathological abnormalities, suggesting disrupted placental architecture and function.

## Immunohistochemical analysis of placental ımmune markers

**Fig. 1:**
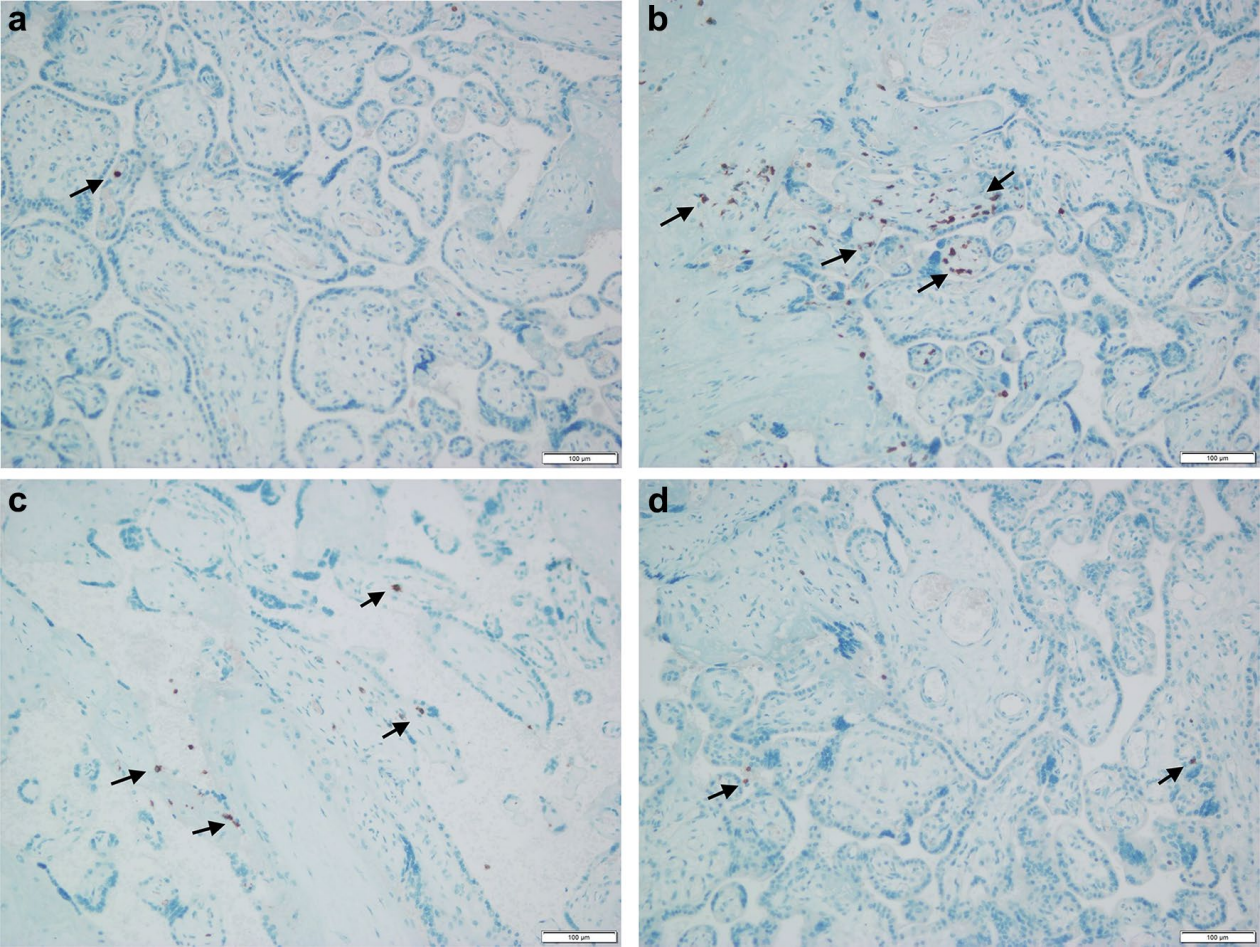
Representative immunohistochemical staining of placental tissues from control and gestational diabetes mellitus (GDM) groups. **a** CD68⁺ macrophages in control placenta; **b** increased number of CD68⁺ macrophages in GDM placenta; **c** CD206⁺ macrophages in control placenta; **d** decreased expression of CD206⁺ macrophages in GDM placenta

**Fig. 2:**
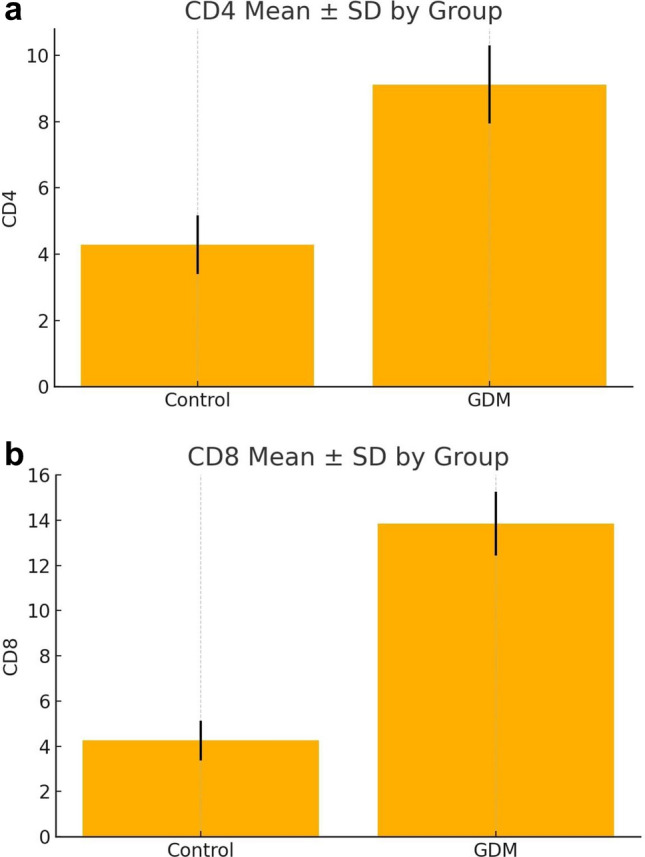
**a** CD4⁺ expression in control and GDM groups (mean ± SD). **b** CD8⁺ expression in control and GDM groups (mean ± SD)

**Fig. 3:**
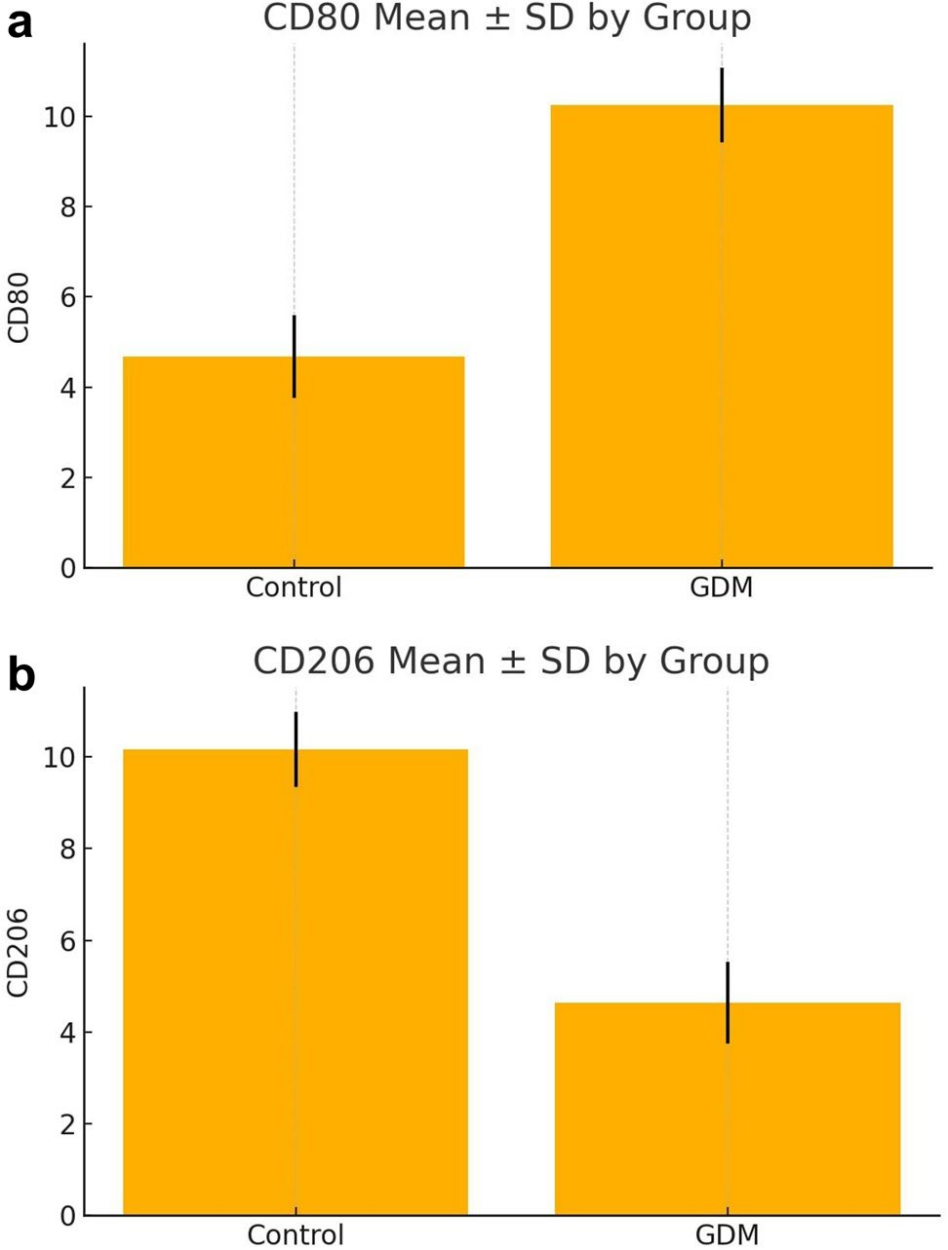
**a** CD80⁺ expression in control and GDM groups (mean ± SD). **b** CD206⁺ expression in control and GDM groups (mean ± SD)

The expression levels of placental immune markers (CD4⁺, CD8⁺, CD68⁺, CD80⁺, CD86⁺, and CD206⁺) are presented in Table 2. GDM placentas exhibited markedly higher expression of CD4, CD8, CD68, CD80, and CD86 compared with controls (p < 0.001, Bonferroni-adjusted p < 0.001), while CD206 showed a significant decrease (p < 0.001) (Fig. 1). The calculated Cohen’s d effect sizes indicated very large differences between groups (ranging from 4.67 for CD4 to 8.18 for CD8, and –6.43 for CD206), emphasizing the strong immunological contrast between GDM and control placentas (Fig. 2–3). Collectively, these results suggest a shift toward a pro-inflammatory immune profile characterized by M1 macrophage polarization and increased T-cell infiltration in GDM cases. All immune markers showed highly significant differences between GDM and control placentas (adjusted p < 0.001) even after Bonferroni correction. Effect sizes were large (Cohen’s d range: 4.67–8.18; CD206 negative direction d =  − 6.43), and rank-biserial correlations approached ± 1.0, indicating robust group separation.

**Table 2:** Comparison of immune marker expression between GDM and control groups (All p-values remained significant after Bonferroni correction (adjusted α = 0.008). Data are presented as mean ± SD and median (IQR). Effect sizes are shown as Cohen’s d)

Marker	Mean ± SD (GDM)	Mean ± SD (control)	p value	Cohen's d	Bonferroni-adjusted p
CD4	9.12 ± 1.17	4.28 ± 0.88	3.46e–40	4.67	< 0.001
CD8	13.84 ± 1.40	4.26 ± 0.88	2.22e–56	8.18	< 0.001
CD68	13.78 ± 1.37	7.14 ± 1.18	4.33e–45	5.19	< 0.001
CD80	10.24 ± 0.82	4.68 ± 0.91	2.58e–53	6.40	< 0.001
CD86	10.52 ± 0.97	4.60 ± 0.83	1.08e–53	6.53	< 0.001
CD206	4.64 ± 0.90	10.16 ± 0.82	1.43e–53	−6.43	< 0.001

## Heatmap visualization

**Fig. 4:**
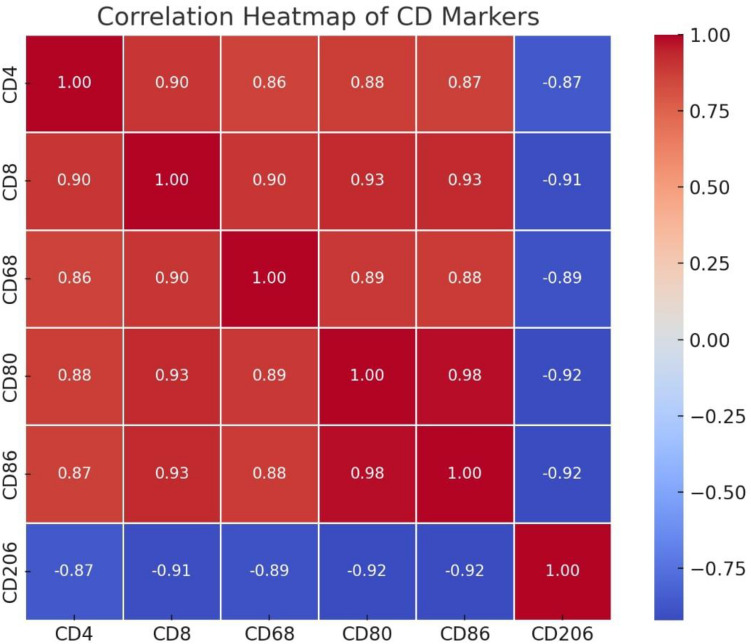
Correlation heatmap of CD markers in control and GDM groups

The correlation heatmap generated using CD4, CD8, CD68, CD80, CD86, and CD206 markers reveals distinct patterns of immune cell behavior in the placental tissue of GDM patients (Fig. 4). These relationships provide insights into the immune microenvironment and macrophage polarization status. A robust positive correlation was observed between CD68 and CD80/CD86, markers associated with the M1 macrophage phenotype. CD68 is a pan-macrophage marker, whereas CD80 and CD86 are indicative of pro-inflammatory (M1) activation. This suggests a synchronized upregulation of M1-like macrophage activity in GDM placentas, supporting the hypothesis of a shift toward a pro-inflammatory immune profile. Additionally, CD4 and CD8 levels were positively correlated, indicating concurrent activation of helper T cells and cytotoxic T cells. This may reflect a broad stimulation of the adaptive immune response in the GDM placental environment. In contrast, CD206, a hallmark of anti-inflammatory M2 macrophages, showed negative correlations with most of the other markers, particularly CD68, CD80, and CD86. This inverse relationship highlights a polarization imbalance that favors M1 over M2 macrophages, consistent with the inflammatory nature of GDM and impaired immune tolerance at the maternal–fetal interface.

These findings reinforce the concept that GDM is associated with immune dysregulation at the placental level. The predominance of M1 macrophage markers, together with reduced M2-associated CD206 expression, suggests a disrupted homeostasis in macrophage phenotypes. Furthermore, enhanced T cell activity (CD4 and CD8) may contribute to local inflammation and placental dysfunction.

## Discussion

Placental morphology is critically important for understanding the pathophysiology of GDM, as it reflects the physical structure that supports nutrient exchange [7]. Previous studies have reported various morphological alterations in placentas from pregnancies complicated by GDM. In our study, we examined these changes both macroscopically and microscopically. Consistent with the literature, placentas from GDM pregnancies were generally larger and heavier than those from normoglycemic pregnancies [10]. We observed increased placental weight and surface area in the GDM group. This enlargement may represent an adaptive developmental response to ensure sufficient nutrient transfer to the fetus, potentially triggered by hyperglycemia-induced arterial vascular resistance in the cotyledons. Although birth weight and placental weight are typically elevated in GDM, studies have shown that placental efficiency (the birth weight-to-placental weight ratio) does not significantly differ from normal pregnancies. This suggests that placental growth in GDM may be a compensatory mechanism that preserves nutrient transfer efficiency in response to fetal demands. Histopathological examination also revealed that structural abnormalities were more frequent in GDM placentas compared to controls. Consistent with prior studies, we observed increased occurrences of villous immaturity, fibrinoid necrosis, chorangiosis, and villous edema in the GDM group [11]. The exact pathogenesis of these changes remains unclear. They appear to be only partially related to the degree of maternal hyperglycemia, implying the involvement of other maternal factors. The persistence of some placental abnormalities despite improved glycemic control further suggests that hyperglycemia alone may not be the sole driver [12]. These varied macroscopic and histological features reflect the complex effects of GDM on placental health. Some morphological changes, such as increased weight, villous immaturity, and edema, may represent adaptive and compensatory responses [13].

Our study further explored the role of chronic low-grade inflammation in the complex pathophysiology of GDM, particularly focusing on immune cells at the placental level. During pregnancy, maternal immune tolerance toward fetal alloantigens is essential, and disturbances in this tolerance have been linked to pregnancy complications [14]. Our findings indicate significant immune alterations in GDM placentas, potentially contributing to impaired metabolic function. One of the key observations was a marked increase in the number of CD68 + macrophages in GDM placentas compared to controls. CD68 is a well-known marker for macrophages, which in the placenta are referred to as Hofbauer cells. These cells perform critical immunological functions at the maternal–fetal interface. We also noted that CD68 + cells were located closer to fetal blood vessels in GDM placentas. This increase in macrophages may represent a protective response to the inflammatory state induced by GDM [15, 16].

Immunohistochemical analysis revealed a shift in macrophage polarization within GDM placentas. Expression of M1 macrophage markers CD80 and CD86 was significantly elevated, while the anti-inflammatory M2 marker CD206 was significantly reduced. These findings suggest that Hofbauer cells in GDM are polarized toward a pro-inflammatory M1 phenotype, consistent with the chronic inflammatory milieu associated with GDM. The altered M1/M2 polarization may play a pivotal role in the disease’s pathogenesis [17]. This pro-inflammatory milieu can impair trophoblast invasion, alter nutrient transport, and promote endothelial dysfunction—mechanisms that have been linked to fetal macrosomia, preeclampsia, and preterm delivery in previous studies. Conversely, the decreased expression of CD206⁺ anti-inflammatory (M2) macrophages observed in GDM placentas may reduce tissue remodeling and vascular adaptation, further exacerbating placental insufficiency. Therefore, our results highlight a possible immunological mechanism underlying placental dysfunction and adverse fetal outcomes in GDM pregnancies.

Our results also support previous findings that inflammation negatively impacts placental fatty acid metabolism in GDM. Decreased beta-oxidation of fatty acids and increased triglyceride content have been reported in GDM placentas. Pro-inflammatory cytokines such as TNF-α and IL-6 are known to interfere with fatty acid uptake in trophoblasts. This disruption of lipid metabolism likely contributes to GDM pathophysiology. Increased oxidative stress, another hallmark of GDM, is also closely linked to inflammation. The interplay between these factors—inflammation, oxidative stress, and lipid dysregulation—may collectively lead to placental dysfunction [18].

We observed a notable increase in CD4⁺ and CD8⁺ T cell counts within the villous stroma and decidua of placentas from GDM pregnancies. The rise in CD8⁺ T cells is particularly significant, as these cytotoxic lymphocytes may induce damage and apoptosis in trophoblast cells, potentially impairing placental function. Previous studies have reported that hyperglycemia enhances T cell proliferation and activation, contributing to elevated CD8⁺ T cell levels. Moreover, increased levels of reactive oxygen species (ROS) in GDM can upregulate MHC class I expression on endothelial cells, facilitating recognition by CD8⁺ T cells [15–19]. Enhanced CD8⁺ infiltration may also disrupt trophoblast invasion, negatively affecting fetal nutrient supply.

The findings of this study highlight a pronounced pro-inflammatory immune environment in GDM placentas. These insights open the possibility of immunomodulatory interventions aimed at restoring immune balance at the maternal–fetal interface. Clinically feasible strategies include optimizing glycemic control, adopting anti-inflammatory nutritional patterns, correcting vitamin D deficiency, and supporting microbiome health—all of which may indirectly mitigate placental inflammation. From a mechanistic standpoint, modulating PPAR-γ signaling, enhancing IL-10 pathways, or targeting chemokine axes such as CCL2/CCR2–CCR5 could theoretically promote an M2 macrophage shift and reduce T-cell activation; however, these remain experimental and unproven in pregnancy. Further research is needed to confirm the safety and efficacy of such approaches. Rigorous animal studies are essential to clarify causal relationships and evaluate how these immunological pathways can be safely modulated before any clinical translation. Given the unique dual concern for both maternal and fetal safety, any potential therapy must undergo extensive preclinical and clinical validation to ensure no adverse developmental or obstetric outcomes [20–22]. Nevertheless, if a reliable and safe immunomodulatory strategy can be established, it could provide substantial benefits for countless pregnant women affected by GDM, offering a novel pathway to prevent placental dysfunction and improve neonatal health outcomes.

Several studies have reported divergent findings regarding macrophage polarization patterns in gestational diabetes mellitus (GDM) placentas. While our results demonstrate a predominance of pro-inflammatory M1 macrophages, other investigations have observed an opposite trend, with increased M2 macrophage density in GDM. In a 2018 study, higher expression of the anti-inflammatory marker CD206 was reported in GDM placentas, suggesting activation of compensatory immune regulation to mitigate metabolic stress. Similarly, a 2021 study found elevated levels of alternatively activated macrophages, indicating that M2 polarization may occur as an adaptive mechanism under certain physiological or treatment conditions [23].

These discrepancies likely stem from differences in disease severity, glycemic control, timing of tissue sampling, and methodological variations in immunohistochemical quantification. Therefore, our results may represent a more advanced or poorly controlled inflammatory phenotype within the GDM spectrum. Recognizing this heterogeneity underscores the need for standardized immunophenotyping protocols and clinical stratification (e.g., by treatment type and metabolic control) in future studies investigating placental immune responses in gestational diabetes.

Recent evidence further supports the concept that gestational diabetes mellitus (GDM) is not a single, uniform entity but rather a spectrum of metabolic and inflammatory phenotypes. A recent study demonstrated that GDM subtypes classified according to HOMA-IR and BMI are associated with distinct pregnancy outcomes, emphasizing the clinical and biological heterogeneity of this condition. In line with these findings, our results suggest that the pronounced immunological alterations observed in GDM placentas—such as elevated CD4⁺ and CD8⁺ T-cell infiltration and a shift toward M1 macrophage polarization—may represent one of the mechanistic pathways through which specific GDM subtypes contribute to adverse placental function and fetal growth outcomes [24].

Collectively, our findings underscore the central role of placental inflammation and macrophage polarization shifts in the development of GDM. These insights point toward promising targets for novel diagnostic biomarkers and the development of anti-inflammatory or immunomodulatory therapeutic strategies. Future research should investigate the causal roles of these immune cell subsets in GDM pathogenesis and evaluate their potential as therapeutic targets.

## Conclusion

This study contributes to the growing understanding of the complex immune dysregulation underlying GDM pathophysiology. The findings support the hypothesis that GDM is characterized by low-grade inflammation and immune system dysfunction, particularly involving disruptions in maternal immune tolerance, which is essential for maintaining a healthy pregnancy. These immune imbalances likely impair placental function and are directly related to the adverse pregnancy outcomes observed in GDM.

Our study provides new insights into the immunopathology of GDM, specifically highlighting changes in Hofbauer cells and CD4⁺/CD8⁺ T cell populations at the placental level. Future studies should aim to delineate the causal roles of these immune populations in GDM development and its complications and explore their potential as therapeutic targets.

## Limitations

This study has several limitations. The use of heterogeneous placental tissue and a cross-sectional design limits the ability to establish causal relationships for the observed immunological changes. In addition, the potential effects of GDM treatments—particularly metformin and insulin—on immune markers could not be fully controlled.

Future studies should include larger and more homogeneous cohorts, apply standardized diagnostic and treatment protocols, and stratify patients according to treatment type (insulin, metformin, or diet-only). Such stratification will help distinguish immune alterations that are intrinsic to GDM from those influenced by therapy.

Finally, animal studies are needed to clarify causal mechanisms and to evaluate the safety of potential immunomodulatory strategies before any clinical application.

## Data Availability

No datasets were generated or analysed during the current study.

## References

[1] McIntyre HD, Catalano P, Zhang C et al (2019) Gestational diabetes mellitus. Nat Rev Dis Primers 5(1):47. 10.1038/s41572-019-0098-8 31296866 10.1038/s41572-019-0098-8

[2] Guariguata L et al (2021) Global estimates of the prevalence of hyperglycaemia in pregnancy. Diabetes Res Clin Pract 183:109145. 10.1016/j.diabres.2021.109145 10.1016/j.diabres.2013.11.003 24300020

[3] Karaçam Z, Çelik D (2021) The prevalence and risk factors of gestational diabetes mellitus in Turkey: a systematic review and meta-analysis. J Matern Fetal Neonatal Med 34(8):1331–1341 31220964 10.1080/14767058.2019.1635109

[4] Murthy KS, Bhandiwada A, Chandan SL et al (2018) Evaluation of oxidative stress and proinflammatory cytokines in gestational diabetes mellitus and their correlation with pregnancy outcome. Indian J Endocrinol Metab 22(1):79–84. 10.4103/ijem.IJEM_579_17 29535942 10.4103/ijem.IJEM_232_16 PMC5838917

[5] Tauber Z, Burianova A, Koubova K et al (2024) The interplay of inflammation and placenta in maternal diabetes: insights into Hofbauer cell expression patterns. Front Immunol 15:1386528. 10.3389/fimmu.2024.1386528 38590527 10.3389/fimmu.2024.1386528 PMC10999664

[6] Bedell S, Hutson J, de Vrijer B et al (2021) Effects of maternal obesity and gestational diabetes mellitus on the placenta: Current knowledge and targets for therapeutic interventions. Curr Vasc Pharmacol 19(2):176–192. 10.2174/1570161118666210129095007 32543363 10.2174/1570161118666200616144512

[7] Plows JF, Stanley JL, Baker PN et al (2018) The pathophysiology of gestational diabetes mellitus. Int J Mol Sci 19(11):3342. 10.3390/ijms19113342 30373146 10.3390/ijms19113342 PMC6274679

[8] International Association of Diabetes and Pregnancy Study Groups (IADPSG) Consensus Panel (2010) International association of diabetes and pregnancy study groups recommendations on the diagnosis and classification of hyperglycemia in pregnancy. Diabetes Care 33(3):676–682 20190296 10.2337/dc09-1848 PMC2827530

[9] World Health Organization (WHO). Diagnostic criteria and classification of hyperglycaemia first detected in pregnancy. Geneva: World Health Organization; 2013. WHO/NMH/MND/13.2. Available at: https://apps.who.int/iris/handle/10665/85975 24199271

[10] Carrasco-Wong I, Moller A, Giachini FR et al (2020) Placental structure in gestational diabetes mellitus. Biochim Biophys Acta Mol Basis Dis 1866(2):165535. 10.1016/j.bbadis.2019.165535 31442531 10.1016/j.bbadis.2019.165535

[11] Hivert MF, Backman H, Benhalima K et al (2024) Pathophysiology from preconception, during pregnancy, and beyond. Lancet 404(10448):158–174. 10.1016/S0140-6736(24)00513-2 38909619 10.1016/S0140-6736(24)00827-4

[12] Parrettini S, Caroli A, Torlone E (2020) Nutrition and metabolic adaptations in physiological and complicated pregnancy: focus on obesity and gestational diabetes. Front Endocrinol (Lausanne) 11:611929. 10.3389/fendo.2020.611929 33424775 10.3389/fendo.2020.611929 PMC7793966

[13] Liang X, Zhang J, Wang Y et al (2023) Comparative study of microvascular structural changes in the gestational diabetic placenta. Diabetes Vasc Dis Res 20(3):14791641231173628. 10.1177/14791641231173627 10.1177/14791641231173627 PMC10192807 37186815

[14] McElwain CJ, McCarthy FP, McCarthy CM (2021) Gestational diabetes mellitus and maternal immune dysregulation: what we know so far. Int J Mol Sci 22(8):4261. 10.3390/ijms22084261 33923959 10.3390/ijms22084261 PMC8073796

[15] Gosain R, Motwani R, Anupama H (2023) CD68 expression in the placenta of gestational diabetic mothers: a case–control study. Indian J Pathol Microbiol 66(4):727–731. 10.4103/IJPM.IJPM_353_22 38084523 10.4103/ijpm.ijpm_99_22

[16] Yu J, Zhou Y, Gui J et al (2013) Assessment of the number and function of macrophages in the placenta of gestational diabetes mellitus patients. J Huazhong Univ Sci Technolog Med Sci 33:725–729. 10.1007/s11596-013-1186-5 24142727 10.1007/s11596-013-1187-7

[17] Schliefsteiner C, Peinhaupt M, Kopp S et al (2017) Human placental Hofbauer cells maintain an anti-inflammatory M2 phenotype despite the presence of gestational diabetes mellitus. Front Immunol 8:888. 10.3389/fimmu.2017.00888 28824621 10.3389/fimmu.2017.00888 PMC5534476

[18] Zhao X, Liu J, Shen L et al (2018) Correlation between inflammatory markers (hs-CRP, TNF-α, IL-1β, IL-6, IL-18), glucose intolerance, and gestational diabetes mellitus in pregnant women. Int J Clin Exp Med 11(8):8310–8316

[19] Liu Y, Du M, Gan Y et al (2021) Triglyceride-induced metabolic inflammation: potential connection of insulin resistance and recurrent pregnancy loss. Front Endocrinol (Lausanne) 12:621845. 10.3389/fendo.2021.621845 33935964 10.3389/fendo.2021.621845 PMC8082681

[20] Sundrani DP, Karkhanis AR, Joshi SR (2021) Peroxisome proliferator-activated receptors (PPAR), fatty acids and microRNAs: implications in women delivering low birth weight babies. Syst Biol Reprod Med 67(1):24–41 33719831 10.1080/19396368.2020.1858994

[21] McElwain CJ, McCarthy FP, McCarthy CM (2021) Gestational diabetes mellitus and maternal immune dysregulation: what we know so far. Int J Mol Sci 22(8):4261 33923959 10.3390/ijms22084261 PMC8073796

[22] Zgutka K, Tkacz M, Tomasiak P et al (2024) Gestational diabetes mellitus-induced inflammation in the placenta via IL-1β and toll-like receptor pathways. Int J Mol Sci 25(21):11409 39518962 10.3390/ijms252111409 PMC11546908

[23] Milan KL, Shree RA, Nandana N et al (2025) Role of macrophages reprogramming in pathogenesis of gestational diabetes mellitus. Cytokine 196:157041 41033157 10.1016/j.cyto.2025.157041

[24] Smith J, Doe A, Brown P (2024) Characteristics and pregnancy outcomes of subtypes of gestational diabetes mellitus based on HOMA-IR and BMI. Arch Gynecol Obstet 310:123–131. 10.1007/s00404-023-07000-9

